# Relationship Between the Nurse–Patient Ratio and Adverse Events in Conventional Hospitalization Units in a Third-Level Hospital

**DOI:** 10.1155/jonm/8885593

**Published:** 2025-11-19

**Authors:** Juan David Fernández-Sánchez, Fernando Andrés-Pretel, Verónica Ortiz-Diaz, Milagros Molina-Alarcón, Cristina M. Lozano-Hernández

**Affiliations:** ^1^Faculty of Nursing of Albacete, University of Castilla-La Mancha (UCLM), Albacete, Spain; ^2^Castilla-La Mancha Health Service (SESCAM), University Hospital Complex of Albacete, Albacete, Spain; ^3^Albacete Integrated Healthcare Management, Castilla-La Mancha Health Service (SESCAM), Albacete, Spain; ^4^Department of Methodology and Statistics, National Paraplegic Hospital Foundation, Toledo, Spain; ^5^Atlantic Mediterranean Technological University, Málaga, Spain; ^6^Coordinator for Quality Management and Improvement, Castilla-La Mancha Health Service (SESCAM), Toledo, Spain; ^7^Institute of Biomedicine (IB-UCLM), University of Castilla-La Mancha (UCLM), Albacete, Spain; ^8^Research Network on Chronicity, Primary Care and Health Promotion (RICAPPS)-(RICORS), Carlos III Health Institute (ISCIII), Madrid, Spain; ^9^Gregorio Marañón Health Research Institute (IiSGM), Center for Biomedical Research Network on Hepatic and Digestive Diseases (CIBEREHD), Madrid, Spain

**Keywords:** adverse events, conventional hospitalization, HAPI, nurse–patient ratio, pressure injuries, workloads

## Abstract

Nurse staffing in hospital inpatient units varies by country and region, highlighting the need for more equitable, evidence-based planning to measure workload based on complexity, thus enabling appropriate staffing in these units. The objective of this study is to determine the relationship between the nurse-to-patient ratio and the incidence of adverse events in adult conventional inpatient units. A descriptive, cross-sectional observational study with an analytical approach was conducted to calculate the relationship between the nurse-to-patient ratio and adverse events in conventional inpatient units at a tertiary care hospital, the Albacete University Hospital Complex (CHUA), from 2018 to 2023, analyzing a total of 113,117 patients. The 24-h median nurse-to-patient ratio was slightly higher in surgical units (11.35) than in medical units (10.79). Spearman's correlation analysis identified significant relationships (rho > 0.4) between the 24-h nurse-to-patient ratio and events such as hospital-acquired pressure injuries (HAPIs), community-acquired pressure injuries, and mortality. However, the multiple linear regression analysis focused on HAPI as the dependent variable, excluding mortality due to its multifactorial nature and community-acquired injuries as they predated admission. The linear regression results showed that for every one-point increase in the 24-h nurse-to-patient ratio, the probability of HAPI increased by 1.94%. Additionally, the probability of HAPI decreased by 2.63% in surgical units compared with medical units. When analyzing the units separately, the relationship was more pronounced in medical units: a one-point increase in the 24-h ratio was associated with a 2.81% increase in the likelihood of HAPI. In surgical units, the 24-h ratio did not show a significant relationship with HAPI, with the patient turnover index being the relevant predictor. The findings confirm that the operational nurse–patient ratios in Spain position the system in a state of room for improvement regarding safety and economic efficiency. The differences observed between medical and surgical units underscore the necessity for staffing policies not to be uniform but rather to become dynamic and contextualized to optimize clinical outcomes and resource management.

## 1. Introduction

Nurses play an indispensable role in any healthcare system, providing continuous, direct, and complex care. A low nurse-to-patient ratio (meaning more nurses per patient) is associated with a reduced workload. This workload is also influenced by factors such as the patient's acute condition, care complexity, administrative tasks, availability of support staff, and interruptions in the clinical environment [1]. Workload is also influenced by factors such as the patient's acuity, the complexity of care, administrative tasks, the availability of support staff, and interruptions in the clinical setting [2].

Currently, different dimensions of care are recognized as measurable, which intrinsically increase the workloads of inpatient nurses [3]. Factors that lead to a greater workload include patient complexity, the adequacy of nurse staffing, unplanned activities, increased documentation, and the patient's assigned specialty (medical or surgical), as well as caring for patients assigned to the unit but originating from other specialties [4, 5].

Additionally, certain key hospital management indicators directly impact the dynamic and demand for care, intensifying the nursing staff's workloads. These include the occupancy rate, the turnover index, the occupancy rate, and the average length of stay [4]. Furthermore, treatment effectiveness, patient satisfaction, patient participation, and patient empowerment, among others, influence nurse organization and workloads [5].

A high workload, staff turnover, organizational and patient complexity factors, the work environment, burnout, lack of time, and the safety culture, irrespective of the nominal ratio, can compromise the nurse's capacity to provide safe, quality care [6, 7]. A greater workload is also related to an increase in adverse events, such as pressure injuries, the incidence of falls, the use of mechanical restraints, missed care, and even mortality [8, 9].

In Spanish hospitals, the method for classifying the complexity of admitted patients is through diagnosis-related groups (DRGs), a system based on the patient's diagnosis and case mix to classify them and evaluate resource consumption, which relates to cost assignment. Patients admitted to conventional inpatient units are classified based on the medical specialty they enter. Adult conventional inpatient units exclude critical care units, intermediate care, obstetrics, and mental health units, distinguishing only between medical and surgical care units. For several years now, adjusted morbidity groups (AMGs), derived from primary care data, have been implemented in Spanish hospitals to measure patient complexity. These groups classify and cluster patients based on their higher complexity and need for care, especially within the context of chronic patient management [10].

Among the adverse events sensitive to nursing care, hospital-acquired pressure injuries (HAPIs) are particularly relevant; they allow for the analysis of not only patient complexity but also the quality and sufficiency of nursing care, given that 95% of them are preventable with adequate nursing management [11]. We can classify HAPI risk factors as intrinsic or patient related (reduced mobility, nutritional status, advanced age, cognitive impairment, skin hydration, and comorbidity) and extrinsic or environment related. Added to these are characteristics of nursing care, such as insufficient staffing, lack of training, and the absence of protocols. Recent research continues to emphasize the multifactorial nature of HAPIs and the importance of comprehensive risk assessment and prevention [2]. Evidence reflects that adequate nurse coverage would be a protective factor against the occurrence of adverse events, indicating that safe coverage is greater than 90% of the required staff [12].

Research by Aiken et al. [13] focuses on adequate nurse staffing and demonstrates that, in surgical units, adding one patient per nurse can increase the 30-day mortality risk by 7%. A more recent study by Lasater et al. [14], conducted in 116 New York hospitals on conventional medical–surgical inpatient floors, with an average ratio of 6.3 patients per nurse, showed that each additional patient increased the probability of in-hospital mortality by 19%, 60-day mortality by 13%, hospital readmission by 6%, and an evident 60% increase in hospital stays Thus, Aiken et al.'s studies highlight the negative consequences of inadequate staffing. However, they have been criticized for not sufficiently addressing economic dimensions. This aspect is considered in the work of Park [15], where a different concept argues that “optimal staffing” and “safe staffing” are not equivalent. This research seeks to achieve the highest quality of nursing care in the most cost-effective manner. This represents a crucial re-evaluation for value-based strategic workforce assessment within a complex healthcare environment. Furthermore, Park explores how AI can guide the safe, ethical, and effective integration of nursing workforce management, utilized to determine acceptable, reasonable, and safe ranges within optimal nursing staffing levels [16].

Internationally, the recognition of the importance of nurse staffing has led to the implementation of various policies. The central debate revolves around two main approaches: fixed ratio mandates and regulated flexibility. States such as California (United States of America) have been pioneers in adopting a policy of strict and legally mandated nurse–patient ratios, which contrasts with Queensland (Australia), which adopted a more flexible approach to implementing staffing policies [17]. Thus, the California model provides a clear and predictable standard, while the Queensland model offers greater flexibility to adapt to the needs of each hospital, achieving better outcomes, such as a 9% reduction in mortality, a 6% reduction in readmissions, and a 3% reduction in average length of stay [17, 18]. Although these policies face detractors, their positive impact on patient safety, quality of care, and the reduction of adverse events is undeniable, even during economic crises. This evidence demonstrates that “safe staffing” is not only a care quality imperative but can also be fiscally optimal in the long term, as investments in personnel are offset by the reduction of costly adverse events [19].

The nurse staffing situation in the Spanish healthcare system contrasts significantly with the optimal practices observed internationally. In Spain, the nurse-per-1000-inhabitants ratio is 6.1–6.3, a figure well below the European Union average, which stands at 8.5. It is estimated that approximately 100,000 additional professionals would be needed to reach this European standard [20].

Furthermore, nurse staffing within Spanish hospitals is far from optimal practices, and its divergence from historical recommendations previously set by the Spanish Ministry of Health is noteworthy [21]. The true reference for establishing safety thresholds in Spain can be found in the 2019 Bill on Nurse Ratios, which, although pending parliamentary procedure, seeks to establish safety limits in healthcare facilities by setting specific thresholds: a maximum of 6–8 patients per nurse in conventional hospitalization [22].

In Spain, the average patient-to-nurse ratio is 1:9 during morning shifts, increasing to 1:13 in the afternoon shift and up to 1:18 in the night shift. The proportions become even greater during weekends, reaching 1:14 in the morning, 1:16 in the afternoon, and 1:19 at night. This territorial variability among Spanish cities is significant, as some provinces have a ratio of less than 8, while others reach more than 10 patients per nurse [23].

Despite the solid international evidence linking adequate nurse staffing with patient safety and the recent legislative efforts in Spain to establish minimum thresholds, national research on this issue lacks a detailed analysis that correlates the variability of ratios by shifts, units, and territories with the occurrence of specific adverse.

A marked knowledge gap persists locally that rigorously and thoroughly quantifies the magnitude of the risk that current nurse–patient ratios pose to the safety of hospitalized patients. This gap prevents evidence-based justification for the implementation of safer and more effective staffing policies in the Spanish context, especially considering that the guidelines established by the Spanish Ministry of Health over 15 years ago are not adequate for the current reality. Therefore, there is a clear need to generate local evidence to drive change [21].

Therefore, it is necessary to sensitize managers beyond a purely quantitative approach, redesigning processes with flexible staffing and integrating technology and interdisciplinary collaboration to achieve an effective staffing policy. The importance of identifying all the dimensions that affect this complex framework must be emphasized in order to offer a theoretical and political approach that is in line with reality.

### 1.1. General Objective

The primary objective of this study was to determine the relationship between the nurse-to-patient ratio and the occurrence of adverse events in adult conventional inpatient units.

### 1.2. Specific Objectives

• To describe hospital management indicators by inpatient unit.
• To analyze the factors associated with the occurrence of HAPI in different care units.

## 2. Methods

### 2.1. Study Design and Setting

An observational, descriptive, cross-sectional study with an analytical approach was conducted. This study utilized Hospital Information System (HIS) and Electronic Health Record (EHR) data from patients admitted to the Albacete University Hospital Complex (CHUA) between 2018 and 2023.

### 2.2. Integration Strategy for Surgical and Medical Wards

The unit of analysis consisted of each of the 13 conventional adult inpatient units, differentiated based on the hospital's physical structure and medical specialties (Table A1). Of these, seven units (A, B, C, D, E, F, and G) were medical care units, accounting for 53.84% of the hospital's total and providing 242 beds. The other six units (a, b, c, d, e, and f) were surgical care units, comprising 46.16% of the hospital's total and offering 195 beds. Critical and intermediate care units, as well as obstetrics and mental health units, were excluded.

### 2.3. Measurements

#### 2.3.1. Adverse Events

The primary outcome variable was adverse events recorded in the patient's EHR, analyzed through HAPIs, community-acquired pressure injuries (acquired prior to admission), hospital falls (falls recorded per 1000 patient-days), and in-hospital mortality (total number of deaths/total discharges ∗ 100).

#### 2.3.2. Hospital Management Indicators

Hospital management indicators included the nurse-to-patient ratio for each work shift: morning (M), afternoon (T), and night (N), as well as a 24-h ratio. Hospitalization indicators comprised occupancy percentage (number of occupied beds/number of available beds ∗ 100), turnover percentage (number of discharges/average available beds ∗ 100), and average length of stay (number of patient-days at discharge/total number of discharges).

### 2.4. Data Collection

The indicators were extracted in an aggregated format from the patient's electronic health record and incorporated from the “Montesinos” dashboard of the Hospital Complex, which is standardized for the entire SESCAM (Castile-La Mancha Health Service).

**Figure 1:**
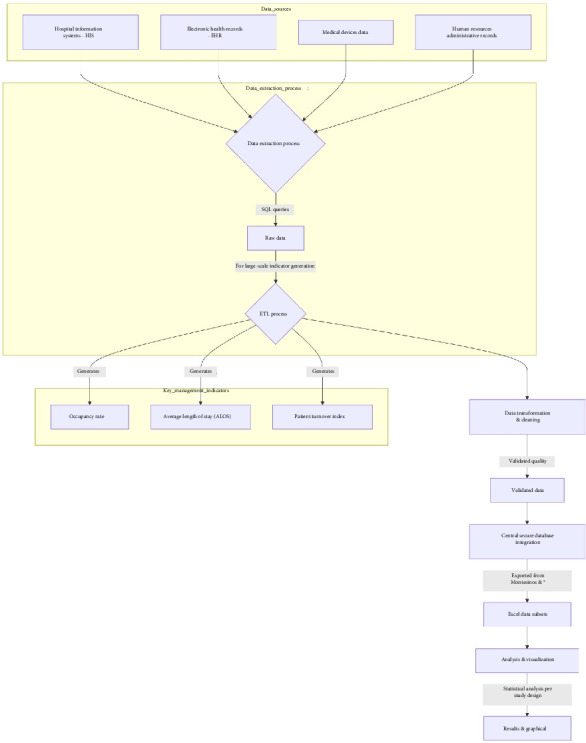
Data integration flowchart.

Data extraction adhered to the General Data Protection Regulation (GDPR), ensuring the anonymization of personal data through its incorporation into a secure and controlled environment standardized for the studied territorial health service (the “Montesinos” dashboard). Data extraction was performed using Structured Query Language (SQL) queries. For the generation of management indicators (occupancy percentage, average length of stay, and turnover rate), the Extract, Transform, Load (ETL) process was utilized. Data consistency and quality were validated during the transformation and cleaning phases. All data were integrated into a central database within a secure environment, providing subsets directly from the Montesinos user interfaces into common formats such as Excel spreadsheets. For the final phase of analysis and visualization, this Excel file was prepared for statistical analysis according to the study design (Figure 1).

The nursing coverage for each inpatient unit was extracted from the Human Resources program of the CHUA.

### 2.5. Statistical Methods

A descriptive analysis of the variables was performed, utilizing absolute frequencies and percentages for categorical data, and measures of central tendency (mean or median) along with measures of dispersion (standard deviation (SD) and interquartile range (IQR)) based on the results of the Kolmogorov–Smirnov normality test.

For the association analysis between variables, Spearman's correlation coefficient was used. For the conclusions to be considered relevant, a rho1 value greater than 0.4 was required, indicating at least a moderate or stronger association. Weaker correlations (weak or very weak) were not considered sufficiently relevant, even if statistically significant in large samples, as they may lack practical impact.

An initial analysis examined the correlation between the 24-h nurse-to-patient ratio and hospital indicators (average length of stay, turnover rate, and occupancy rate), as well as adverse events in the care units, both collectively and separately for medical and surgical care units.

For linear regression analysis, HAPI was included as the dependent variable and the 24-h nurse-to-patient ratio as the independent variable. Additionally, turnover rate and average length of stay were included in the regression model to control for and evaluate the influence of patient flow dynamics on outcomes in each care unit. The influence of the nurse-to-patient ratio on the occurrence of HAPI was also considered. A multiple linear regression using a saturated model was performed on the care units both jointly and separately.

All analyses were conducted using R software (v.4.2), with an alpha error of 0.05 and a 95% confidence interval.

## 3. Results

The study sample analyzed 113,117 patients discharged from the inpatient units under investigation. Of these, 55.66% were men, and the median age was 63.48 years (IQR: 7.84).

Table 1 presents the 24-h nurse-to-patient ratios for the various hospitalization floors, detailing hospital management indicators by care unit (medical vs. surgical).

Overall, the median 24-h nurse-to-patient ratio was higher in surgical care units than in medical care units (10.79, IQR: 2.18 and 11.35, IQR: 0.93, respectively). The night shift consistently had the highest ratio in both types of units, followed by the afternoon and morning shifts. Regarding the median occupancy rate, it was slightly higher in medical care units (82.32, IQR: 9.24: vs. 81.61, IQR: 11.72), as was the average length of stay (7.54, IQR: 1.88 vs. 4.89, IQR: 1.20). However, the turnover rate was lower in medical units (3.32, IQR: 0.97 vs. 5.17, IQR: 1.11).

The overall mortality rate for all these units had a median of 3.28 (IQR: 7.05). Specifically, the median mortality in medical care units was 7.36 (IQR: 11.49), whereas for surgical care units, it was 1.87 (IQR: 2.38). For HAPIs, the median in medical care units was 4.19 (IQR: 8.12), while in surgical care units, the median was 1.57 (IQR: 3.35).

**Figure 2:**
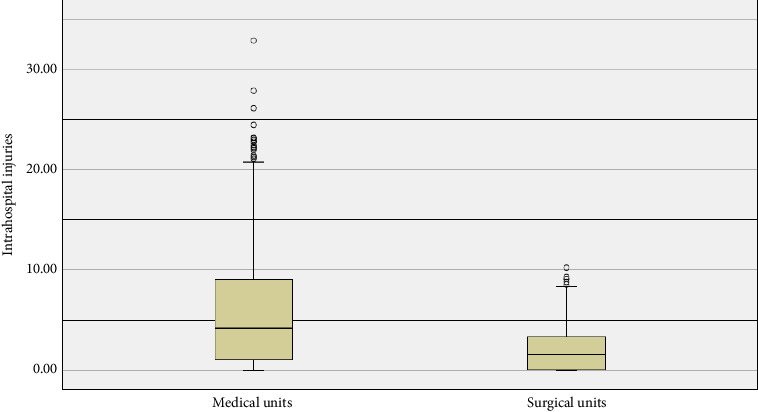
Prevalence of hospital-acquired pressure injuries.

Regarding HAPI across all care units (including both medical and surgical), the median was 2.34 (IQR: 5.05). For community-acquired pressure injuries (those occurring prior to admission), the median was 0.94 (IQR: 3.24), with both showing a *p* < 0.001.  Figure 2 illustrates the comparison of HAPI prevalence between medical and surgical care units.

We can also observe that some patients admitted to the various units already present with a pressure injury (community acquired). This is more prevalent in medical care units (median: 2.08 and IQR: 5.60) compared with surgical care units (median: 0.71 and IQR: 1.53).

### 3.1. Correlation Analysis

The correlation study between nurse-to-patient ratios and adverse events did not show relevant results when analyzing all care units together (Table 2 and Table A2).

Conversely, the correlation study in medical care units, shown in Table 3, indicates that the 24-h nurse-to-patient ratio is directly associated with: HAPI (rho: 0.491; *p* < 0.001), community-acquired pressure injuries (rho: 0.476; *p* < 0.001), mortality rate (rho: 0.533; *p* < 0.001), and more weakly with average length of stay (rho: 0.217; *p* < 0.001). As the 24-h average ratio increases, the percentage of HAPI and mortality also increase (Table A3).

When we performed the correlation analysis of the 24-h nurse-to-patient ratio with the previously studied indicators in surgical care units, it did not show a relevant association for the study (Table 4 and Table A4).

A specific study of the correlation between the 24-h nurse-to-patient ratio and HAPI revealed a correlation of 0.07 (nearly negligible) in the overall sample (analyzing all units). However, this correlation increased to 0.49 (moderate) within medical units, while presenting a weak inverse rho value of −0.33 in surgical units. The difference in rho values between medical and surgical units was statistically significant (*p* < 0.001) (Table A5).

#### 3.1.1. Linear Regression Analysis

Table 2 shows a negative impact of the ratio: a higher number of patients per nurse correlates with a greater incidence of HAPI, even when considering all other studied factors. Regarding the factors associated with HAPI development, the probability of experiencing HAPI increased by 1.94% for every point increase in the 24-h ratio. It also increased by 1.42% for every point decrease in the turnover rate (Table 5). Conversely, the probability of developing HAPI decreased by 2.63% in surgical inpatient units compared with medical care units.

The average length of stay did not yield significant results in the final model. Therefore, we will not include it in subsequent regressions.

When performing the following linear regressions, we will analyze the medical and surgical units separately, as this allows for a more precise and contextualized analysis of the relationship between the nurse-to-patient ratio and HAPI prevalence.

The regression analysis, including only medical care units, showed that the percentage of HAPI continued to be statistically significant when correlated with the 24-h ratio, with a coefficient of 2.81 (*p* < 0.001) and a turnover rate of −1.34 (*p* < 0.001). Therefore, in medical care units, a one-point increase in the 24 h nurse-to-patient ratio increases the probability of having HAPI by 2.81%.

The unit with the greatest impact is U.F., where the coefficient is 7.90 (*p* < 0.001). The only unit that did not achieve statistical significance is U.G., with *p*=0.218, which could be explained by it being a unit that includes palliative care with a higher mortality rate (as reflected in Table 1) compared with the others, see Table 6.

In the detailed analysis of surgical care units, the 24-h ratio did not show a relationship with HAPI. However, the turnover rate did, with a coefficient of 0.31 (*p* < 0.001). The only unit where the turnover rate correlated with HAPI occurrence was Unit d, with a coefficient of 2.21 (*p* < 0.001). This might be explained by Unit d having the lowest turnover rate among all surgical care units, as reflected in Table 1; see Table 7.

## 4. Discussion

The present study focuses on analyzing the relationship between the nurse–patient ratio and the occurrence of adverse events sensitive to nursing care, specifically in-hospital mortality and the prevalence of Hospital-Acquired Pressure Injuries. This work addresses a fundamental gap existing in the Spanish healthcare literature by quantifying, through specific correlations, the risk of suboptimal staffing in hospitalization units, thereby providing the necessary empirical evidence to justify urgent regulatory reform in a country that lacks legislated minimum nurse ratios.

Its main findings show that deficient staffing strongly and significantly correlates with an increase in in-hospital mortality (rho: 0.533; *p* < 0.001) and with a higher incidence of HAPIs (rho: 0.491; *p* < 0.001). These results position nurse staffing not as a secondary factor but as a structural determinant of safety and care quality within the Spanish National Health System.

The analysis of the patient-to-nurse ratios in the hospitalization units that are the focus of this study reflects a reality in care delivery that, while it may appear slightly better than previous data, remains deeply compromised with respect to international standards of safe practice. The figures obtained in this work (1:8, 1:10, and 1:13) are slightly lower than those recorded in the 2019 study by Lendínez et al. [23]. For example, in the morning shift, the ratio improved from 1:9 to 1:8 and in the night shift, from 1:18 to 1:13. Despite this apparent trend toward improvement compared with previous Spanish studies, a critical analysis reveals that this variation is irrelevant to patient safety. When compared with the clinical safety threshold, the Spanish context remains in a state of structural safety deficit.

Scientific evidence, such as that from Havaei et al. [24], establishes that the inflection point where care quality begins to deteriorate, safety is compromised, and staff burnout escalates, which is found between the 1:5 and 1:6 ratio. The ratios observed in the present study, even in the best-staffed shift (morning: 1:8), are well above this limit. This provides the clearest empirical framework for assessing the intrinsic risk of the Spanish ratios.

The most significant and revealing contrast for Spanish health policy is established when comparing the results with contexts where staffing is legislated, such as Australia. In that country, the study by McHugh et al. [25] analyzed the patient-to-nurse ratio in 68 public hospitals and found a 24-h average ratio of 1:5 in surgical units. In contrast, the surgical units analyzed in the present study showed a ratio of 1:11, more than double the number of patients per nurse than the Australian average. The Australian methodology, consistent with its legislation, focuses on the compliance of legally mandated minimum staffing averages. The difference of 1:11 versus 1:5 illustrates that the lack of legislation in Spain not only results in poor ratios but also institutionalizes the failure in clinical surveillance by drastically reducing the time available per patient.

The correlation found in this study between nurse staffing and in-hospital mortality (rho: 0.533; *p* < 0.001) is a powerful indicator that personnel management has a direct impact on patient survival. This conclusion aligns with international evidence, confirmed by the meta-analysis by Gonete [26] and the study by Cordina et al. [27], which reflects the protective effect of safe nurse staffing, associating it with a reduction of nearly 60% in the risk of mortality (RR: 0.41 and 95% CI: 0.37–0.45).

The direct correlation found between the nurse–patient ratio and the prevalence of HAPIs (rho: 0.491; *p* < 0.001) links the staffing deficit with one of the most sensitive and costly indicators of preventable morbidity. The results of this work coincide with the study by Juvé-Udina et al. [12], which unequivocally links adequate nursing staffing levels to the prevention and reduction of HAPI, with an RR of 0.69 (95% CI: 0.67–0.72). They also coincide with the study by Peng et al. [28] on the impact of nurse staffing, turnover, and quality, which concludes that nurse staffing is negatively associated with the HAPI rate in medical–surgical units. HAPIs, as reflected in the study by Serafin et al. [29], affect millions of people annually and contribute to tens of thousands of deaths in countries like the United States and the United Kingdom. They are widely considered the most common preventable adverse event in hospitals and are classified as “never events” by the U.S. Centers for Medicare and Medicaid Services; tragically, however, they cause approximately 60,000 patient deaths annually in the United States alone [30, 31].

The Sweet Spot Theory by Park [32] proposes a safe staffing level that challenges the conventional assumption that indiscriminately increasing nurse staffing always results in better outcomes. Instead, it advocates for optimization to achieve a “Central Optimal Nurse Staffing Zone” (C-ONSZ), defined as the critical intersection where a balance is achieved among patient safety (positive clinical outcomes), staffing sufficiency (lower burnout and turnover), and economic efficiency (maximizing health outcomes relative to cost). The present study provides the necessary empirical evidence to position Spanish hospital units outside this Central Optimal Staffing Zone, given that the current ratios (1:8–1:13) are above the clinical safety threshold (1:6) and correlate directly with worse safety outcomes (mortality rho: 0.533 and HAPI rho: 0.491) [24].

This indicates that the units are operating in the dangerously low staffing quadrant (Unsafe), where safety is critical. There is also a failure in economic efficiency; the high correlation with HAPI (rho: 0.491) demonstrates that the current staffing is not efficient and that the costs derived from complications (45% of the cost of HAPIs due to extra length of stay) exceed the initial investment in personnel [33]. The theory by Park [32] justifies strategic investment to move staffing toward the C-ONSZ, where the benefits in clinical outcomes and cost savings offset the increase in workforce.

The Sweet Spot must also be dynamic. The present study ratifies this by finding a relationship between the patient turnover index and the occurrence of HAPIs, a finding that underscores the necessity for dynamically adjusting staffing according to the studies by Park et al. [15, 34]. In hospitalization units, the beneficial effect of adequate nurse staffing was reduced by 11.5% when patient turnover increased [34]. This reinforces that staffing which appears adequate in terms of the patient-to-nurse ratio can be functionally suboptimal if it is not adjusted to the high volume of admissions, discharges, and transfers, which dilutes the positive impact of the personnel [15].

## 5. Study Limitations

The primary unit of analysis in our research was hospitalization services, not individual patients or nurses. This means that variability in the clinical practice of specific nurses (e.g., the exceptional skill or dedication of a professional) could have masked the true impact of an unfavorable nurse-to-patient ratio at the individual level. It is possible that the efforts of highly trained and committed nurses mitigated the consequences of insufficient staffing at certain times or units, or, conversely, that the negative impact of low ratios was less apparent if some nurses were unable to compensate for the overload.

Another significant limitation lies in the classification of care units. The dynamics of bed management within the CHUA make a strict differentiation between medical and surgical units challenging. We observed that a small percentage of surgical patients were admitted to medical units and vice versa.

The absence of a direct measure of patient complexity in our study represents a significant limitation that could influence the interpretation of our findings regarding the relationship between nurse staffing and adverse events. Patient complexity is a key determinant of nursing workload, as more complex patients require a greater amount of time, skills, and resources from nursing staff, regardless of the gross patient-to-nurse ratio.

We recognize that patient complexity is a crucial factor, and its full consideration would be ideal for understanding the staffing–outcome relationship. In this study, complexity could not be measured through the AMGs, as this system was implemented in our Hospital Complex in 2024. Therefore, this study focuses on staffing and the importance of adequately adjusting staffing in inpatient units [35, 36]. Given the lack of a direct measure of this variable, it is recommended to interpret our findings within this context and consider patient complexity as a priority for future research, as our Health Service is implementing a new patient classification system based on complexity, which will open interesting opportunities for future research with a greater level of detail.

It is worth noting the comparability of HAPI results between each of the studied care units, as both medical and surgical units utilize a clinical practice guideline for pressure injury prevention approved and implemented at CHUA [37].

## 6. Conclusions and Implications for Nursing Management

The study of the relationship between the nurse–patient ratio and the occurrence of adverse events in a tertiary Spanish hospital has generated evidence for nursing management and Spanish health policy, showing an inverse relationship between the nurse–patient ratio and the occurrence of adverse events in conventional adult hospitalization units. The surgical care units showed a lower nurse–patient ratio than the medical care units.

Regarding the hospital management indicators, in the surgical care units, the patient turnover index was the most critical factor, correlating inversely with the probability of HAPI prevalence.

The medical care units, which presented a higher prevalence of adverse events (HAPIs, community-acquired pressure injuries, incidence of falls, and mortality), showed a direct relationship between lower nurse staffing and the prevalence of HAPIs, as well as mortality.

The findings confirm that the operational ratios in Spain (1:8–1:13) position the system in a state of room for improvement regarding safety and economic efficiency.

The results of this study underscore the urgent need to implement safe and adequate nurse staffing policies. This is crucial not only for patient safety (by reducing mortality and HAPIs) but also for the sustainability of the health system and staff well-being.

Nursing managers and health policy must prioritize Park's Sweet Spot Theory to move from simple recruitment to strategic optimization. Implementing a maximum limit of six patients per nurse, along with a dynamic system that adjusts staffing to the patient turnover index (especially in surgical units), is essential for reaching C-ONSZ, where safety, workforce sufficiency, and economic efficiency are balanced.

The differences observed in the correlations between medical and surgical units underscore that staffing policies must not be uniform. It is essential that these policies adapt to the specific needs of each unit type, considering factors such as patient complexity, occupancy, average length of stay, and the turnover index, as required by dynamic workload models.

The prioritization of adequate staffing must be accompanied by strategies that improve the work environment and the safety climate, as this directly reduces mortality and mitigates the occupational stress that drives turnover intention. Managers must use these data to negotiate and justify the necessary resources for nursing personnel, understanding the investment not as a cost but as the main talent retention tool for the Spanish National Health System.

## Figures and Tables

**Table 1:** Hospital management indicators and adverse events associated with nurse staffing ratios by type of care unit.

	Medical care units								Surgical care units						
	U.B (4)	U.B (5)	U.C (6)	U.D (9)	U.E (11)	U.F (12)	U.G (14)	Total (median and IQR)	U.a (2)	U.b (3)	U.c (7)	U.d (8)	U.e (10)	U.f (13)	Total (median and IQR)
Ratio 24 h	11.33 (0)	10.51 (0.55)	10.51 (1.71)	9.92 (0)	8.54 (0)	12.30 (0.41)	12.30 (0.41)	10.79 (2.18)	10.79 (0)	11.72 (0)	11.35 (0)	10.79 (0)	11.35 (0)	14.17 (0)	11.35 (0.93)
Ratio M	8 (0)	7.80 (0.40)	8 (0)	7.30 (0)	7.50 (0)	8.60 (0)	8.60 (0)	8 (1.1)	8 (0)	8.6 (0)	7.5 (0)	8 (0)	7.5 (0)	10 (0)	8 (1.1)
Ratio T	8 (0)	9.75 (0.50)	9.75 (2)	11 (0)	7.5 (0)	9.30 (1.40)	9.30 (1.40)	9.05 (2)	10 (0)	13 (0)	10 (0)	10 (0)	10 (0)	10 (0)	10 (0)
Ratio N	16 (0)	12.95 (0.55)	12.95 (2.70)	11 (0)	10 (0)	17 (0)	17 (0)	13.30 (6)	13.30 (0)	13 (0)	15 (0)	13.30 (0)	15 (0)	20 (0)	13.30 (1.70)

Occupancy rate	84.31 (7.37)	91.10 (6.40)	84.68 (17.42)	81.17 (5.81)	79.29 (8.17)	82.45 (4.73)	79.40 (8.09)	82.32 (9.24)	82.39 (5.92)	86.23) (6.78)	85.74 (8.96)	81.86 (5.70)	79.11 (6.58)	45.22 (15.39)	81.61 (11.72)
Bed turnover rate	3.56 (0.75)	3.57 (0.56)	3.44 (0.70)	5.38 (0.87)	2.89 (0.48)	2.98 (0.40)	2.96 (0.47)	3.32 (0.97)	5.08 (0.55)	5.27 (0.53)	4.95 (0.81)	3.65 (0.71)	5.42 (0.73)	9.48 (2.35)	5.17 (1.11)
Average length of stay (days)	7.02 (1.24)	7.46 (1.23)	8.98 (2.95)	4.62 (0.63)	8.05 (2.05)	8.25 (0.95)	8.28 (1.35)	7.54 (1.88)	4.86 (0.53)	5 (0.57)	5.20 (0.72)	6.73 (1.09)	4.46 (0.54)	1.43 (0.22)	4.89 (1.20)

Hospital-acquired pressure injuries (HAPIs)	3.31 (5.11)	2.77 (3.97)	6.67 (4.73)	1.24 (2.33)	2.47 (4.86)	16.54 (8.25)	9.27 (4.48)	4.19 (8.12)	1.81 (3.12)	2.28 (2.61)	1.52 (1.81)	4.42 (4.51)	1.20 (2.14)	0 (0.96)	1.57 (3.35)
Community-acquired pressure injuries	2.08 (4.22)	0.89 (2.06)	3.91 (3.70)	0 (0.79)	1.60 (3.04)	12.88 (7.79)	4.51 (4.76)	2.08 (5.60)	0.64 (1.31)	0.75 (1.44)	0.68 (0.94)	2.20 (2.53)	0.69 (1.26)	0 (0.90)	0.71 (1.53)
Falls in hospitals	0 (2.19)	0.96 (1.89)	1.18 (2.33)	1.18 (1.47)	1.55 (2.99)	0.74 (0.84)	0.86 (1.61)	0.96 (2.07)	0 (1.15)	0 (1.33)	0 (0.98)	0.96 (1.53)	0 (1.25)	0 (0)	0 (1.15)
Mortality rate	3.28 (3.29)	5.04 (4.01)	8.39 (5.08)	1.42 (1.33)	7.80 (3.99)	17.16 (4.54)	23.34 (7.22)	7.36 (11.49)	2 (1.61)	1.16 (2.52)	2.66 (1.85)	2.85 (2.48)	2.42 (2.30)	0 (0.50)	1.87 (2.38)

**Table 2:** Correlogram of ratios and adverse events across the set of care units.

		Intrahospital injuries	Community-acquired injuries	Hospital falls	Mortality rate	Medium stay	Rotation index	Occupation index	Nurse–patient ratio 24 h
Intrahospital injuries	rho	1	0.682	0.115	0.541	0.509	−0.532	0.059	0.069
	*p*		< 0.001	< 0.001	< 0.001	< 0.001	< 0.001	0.068	0.035

Community-acquired injuries	rho		1	0.083	0.523	0.457	−0.464	0.043	0.106
	*p*			0.011	< 0.001	< 0.001	< 0.001	0.186	0.001

Hospital falls	rho			1	0.183	0.208	−0.223	0.034	−0.242
	*p*				< 0.001	< 0.001	< 0.001	0.288	< 0.001

Mortality rate	rho				1	0.693	−0.712	0.047	0.007
	*p*					< 0.001	< 0.001	0.150	0.807

Medium stay	rho					1	−0.93	0.269	−0.165
	*p*						< 0.001	< 0.001	< 0.001

Rotation index	rho						1	0.003	0.207
	*p*							0.919	< 0.001

Occupation index	rho							1	−0.096
	*p*								0.003

Nurse–patient ratio 24 h	rho								1
	*p*								

**Table 3:** Correlogram of nurse-to-patient ratios and adverse events across medical care units.

		Intrahospital injuries	Community-acquired injuries	Hospital falls	Mortality rate	Medium stay	Rotation index	Occupation index	Nurse–patient ratio 24 h
Intrahospital injuries	rho	1	0.757	−0.042	0.562	0.281	−0.347	−0.114	0.491
	*p*		< 0.001	0.340	< 0.001	< 0.001	< 0.001	0.010	< 0.001

Community-acquired injuries	rho		1	−0.057	0.569	0.262	−0.29	−0.068	0.477
	*p*			0.197	< 0.001	< 0.001	< 0.001	0.123	< 0.001

Hospital falls	rho			1	−0.031	−0.011	−0.037	−0.066	−0.217
	*p*				0.475	0.796	0.397	0.137	−0.223

Mortality rate	rho				1	0.507	−0.567	−0.211	0.533
	*p*					< 0.001	< 0.001	< 0.001	< 0.001

Medium stay	rho					1	−0.836	0.080	0.217
	*p*						< 0.001	0.071	< 0.001

Rotation index	rho						1	0.373	−0.131
	*p*							< 0.001	0.003

Occupation index	rho							1	0.116
	*p*								0.008

Nurse–patient ratio 24 h	rho								1
	*p*								

**Table 4:** Correlogram of nurse-to-patient ratios and adverse events across surgical care units.

		Intrahospital injuries	Community-acquired injuries	Hospital falls	Mortality rate	Medium stay	Rotation index	Occupation index	Nurse–patient ratio 24 h
Intrahospital injuries	rho	1	0.498	0.108	0.366	0.501	−0.52	0.207	−0.332
	*p*		< 0.001	0.024	< 0.001	< 0.001	< 0.001	< 0.001	< 0.001

Community-acquired injuries	rho		1	0.081	0.264	0.386	−0.387	0.112	−0.282
	*p*			0.090	< 0.001	< 0.001	< 0.001	0.019	< 0.001

Hospital falls	rho			1	0.144	0.178	−0.19	0.123	−0.158
	*p*				0.002	< 0.001	< 0.001	0.010	< 0.001

Mortality rate	rho				1	0.408	−0.438	0.237	−0.355
	*p*					< 0.001	< 0.001	< 0.001	< 0.001

Medium stay	rho					1	−0.879	0.533	−0.499
	*p*						< 0.001	< 0.001	< 0.001

Rotation index	rho						1	−0.220	0.600
	*p*							< 0.001	< 0.001

Occupation index	rho							1	−0.154
	*p*								0.001

Nurse–patient ratio 24 h	rho								1
	*p*								

**Table 5:** Factors associated with the development of hospital-acquired pressure injuries in medical and surgical care units: multiple linear regression.

	**Initial model**				**Final model**			
	**Coef. (IC 95%)**	**SE**	**Valor t**	**p value**	**Coef. (IC 95%)**	**SE**	**t-value**	**p value**

Ratio 24 h	1.908 (1.680, 2.136)	0.116	16.433	< 0.001	1.938 (1.714, 2.161)	−0.113	17.026	< 0.001
Bed turnover rate	−1.255 (−1.572, −0.938)	0.161	−7.782	< 0.001	−1.425 (−3.245, −2.027)	0.089	−16.006	< 0.001
Average length of stay	0.183 (−0.101, 0.469)	0.145	1.263	0.206	—			—

Type of hospitalization								

**Medical**	**Ref.**				**Ref.**			

Surgical	−2.464 (−3.129, −1.798)	0.339	−7.267	< 0.001	−2.636 (−1.600, −1.250)	0.339	−7.267	< 0.001
*R* ^2^	0.397			< 0.001	0.397			< 0.001
Adjusted *R*^2^	0.394			< 0.001	0.394			< 0.001

*Note:* HAPI ∼ 24-h nurse-to-patient ratio + type of hospitalization + bed turnover rate + average length of stay.

**Table 6:** Factors associated with the development of hospital-acquired pressure injuries in medical care units: multiple linear regression.

	**Initial model**				**Final model**			
	**Coef. (IC 95%)**	**SE**	**Valor t**	**p value**	**Coef. (IC 95%)**	**SE**	**Valor t**	**p value**

Ratio 24 h	2.815 (1.768, 3.862)	0.532	5.283	< 0.001	2.810 (1.768, 3.862)	−0.532	5.283	< 0.001
Bed turnover rate	−1.345 (−2.063, −0.628)	0.365	−3.687	< 0.001	−1.345 (−2.063, −0.628)	0.365	−3.687	< 0.001
Medical units								
Unit A	Ref				Ref.			
Unit B	2.134 (0.523, 3.745)	0.820	2.603	0.009	2.134 (0.523, 3.745)	0.820	2.603	0.009
Unit C	6.444 (4.470, 8.418)	1.004	6.415	< 0.001	6.444 (4.470, 8.418)	1.004	6.415	< 0.001
Unit D	4.080 (1.560, 6.600)	1.282	3.181	< 0.001	4.080 (1.560, 6.600)	1.282	3.181	< 0.001
Unit E	6.379 (3.215,9.543)	1.610	3.962	< 0.001	6.379 (3.215, 9.543)	1.610	3.962	< 0.001
Unit F	7.907 (6.115, 9.700)	0.912	8.667	< 0.001	7.907 (6.115, 9.700)	0.912	8.667	< 0.001
Unit G	1.137 (−0.675, 2.951)	0.923	1.233	0.218				
*R* ^2^	0.533			< 0.001	0.533			< 0.001
Adjusted *R*^2^	0.526			< 0.001	0.526			< 0.001

*Note:* HAPI ∼ 24-h nurse-to-patient ratio + medical units + bed turnover rate.

**Table 7:** Factors associated with the development of hospital-acquired pressure injuries in surgical care units: multiple linear regression.

	Initial model				Final model			
	Coef. (IC 95%)	SE	*t*-value	*p* value	Coef. (IC 95%)	SE	*t*-value	*p* value
Ratio 24 h	0.121 (−0.196, 0.439)	0.161	0.750	0.453				
Bed turnover rate	−0.337 (−0.521, −0.152)	0.093	−3.593	< 0.001	0.315 (−0.490, −0.140)	0.089	−3.538	< 0.001
Surgical units								
Unit a	Ref.				Ref.			
Unit b	0.226	0.332	0.682	0.495				
Unit c	−0.295	0.314	−0.940	0.347				
Unit d	2.096 (1.448, 2.746)	0.329	6.364	< 0.001	2.218 (1.486, 2.770)	0.326	6.521	< 0.001
Unit e	−0.344	−0.311	−1.105	0.269				
Unit f	−0.163	0.599	−0.273	0.784				
*R* ^2^	0.325			< 0.001	0.324			< 0.001
Adjusted *R*^2^	0.313			< 0.001	0.314			< 0.001

*Note:* HAPI ∼ 24-h nurse-to-patient ratio + surgical units + bed turnover rate.

**Table 8:** Functional care units.

Care medical		Care surgical	
U.A.	Nephrology	U.a.	Traumatology
U.B.	Digestive and infectious diseases	U.b.	Hemodynamics and cardiology tests
U.C.	Internal medicine and pulmonology	U.c.	General and thoracic surgery
U.D.	Neurology	U.d.	General and vascular surgery
U.E.	Oncology and hematology	U.e.	Urology and gynecology
U.F.	Geriatrics	U.f.	Multispecialty surgical unit
U.G.	Internal medicine and palliative care		

**Table 9:** Correlation between nurse–patient ratios in 24 h with different numerical variables related to adverse events.

Parameter 1	Parameter 2	Rho	CI	CI_low	CI_high	*S*	*p*
RATIO_24H	Hospital-acquired injuries	0.0687594	0.95	0.0028048	0.1341184	127,273,411	0.248
RATIO_24H	Community-acquired injuries	0.1057302	0.95	0.0400422	0.1705083	122,220,581	0.011
RATIO_24H	Hospital falls	−0.2415741	0.95	−0.3027174	−0.1784505	169,686,951	< 0.001
RATIO_24H	Mortality rate	0.0109039	0.95	−0.0614103	0.0831042	78,229,461	< 0.001
RATIO_24H	Average stay	−0.1650149	0.95	−0.2284950	−0.1001376	159,223,544	< 0.001
RATIO_24H	Rotation index	0.2074726	0.95	0.1434688	0.2697480	108,315,373	< 0.001
RATIO_24H	Occupation index	−0.0956397	0.95	−0.1605939	−0.0298608	149,741,980	0.030

**Table 10:** Correlation between nurse–patient ratios in 24 h with different numerical variables related to adverse events in medical care units.

Parameter 1	Parameter 2	Rho	CI	CI_low	CI_high	*S*	*p*
RATIO_24H	Hospital-acquired injuries	0.4914771	0.95	0.4201323	0.5567836	10,850,484	< 0.001
RATIO_24H	Community-acquired injuries	0.4768492	0.95	0.4042717	0.5434591	11,162,605	< 0.001
RATIO_24H	Hospital falls	−0.2174225	0.95	−0.3014398	−0.1300550	25,976,461	< 0.001
RATIO_24H	Mortality rate	0.5335121	0.95	0.4591428	0.6004555	5,760,161	< 0.001
RATIO_24H	Average stay	0.2168073	0.95	0.1294202	0.3008527	16,711,185	< 0.001
RATIO_24H	Rotation index	−0.1313063	0.95	−0.2186351	−0.0418909	24,138,976	0.034
RATIO_24H	Occupation index	0.1164279	0.95	0.0267985	0.2042003	18,853,007	0.088

**Table 11:** Correlation between nurse–patient ratios in 24 h with different numerical variables related to adverse events in surgical care units.

Parameter 1	Parameter 2	Rho	CI	CI_low	CI_high	*S*	*p*
RATIO_24H	Hospital-acquired injuries	−0.3317903	0.95	−0.4155194	−0.2424855	17,895,074	< 0.001
RATIO_24H	Community-acquired injuries	−0.2818234	0.95	−0.3688464	−0.1899027	17,223,676	< 0.001
RATIO_24H	Hospital falls	−0.1579720	0.95	−0.2512359	−0.0618018	15,559,502	< 0.001
RATIO_24H	Mortality rate	−0.3948558	0.95	−0.4810417	−0.3011109	10,846,315	< 0.001
RATIO_24H	Average stay	−0.4988535	0.95	−0.5684326	−0.4221893	20,139,879	< 0.001
RATIO_24H	Rotation index	0.5997035	0.95	0.5336671	0.6584710	5,378,727	< 0.001
RATIO_24H	Occupation index	−0.1538217	0.95	−0.2472465	−0.0575633	15,503,736	0.008

**Table 12:** Comparison of correlations at the sample level, medical, and surgical care units.

Parameter 1	Parameter 2	r_sample	r_med	r_surg	p_sample	p_med	p_surgi	p.comp
RATIO_24H	Hospital-acquired injuries	0.0687594	0.4914771	−0.3317903	0.248	< 0.001	< 0.001	< 0.001
RATIO_24H	Community-acquired injuries	0.1057302	0.4768492	−0.2818234	0.011	< 0.001	< 0.001	< 0.001
RATIO_24H	Hospital falls	−0.2415741	−0.2174225	−0.1579720	< 0.001	< 0.001	0.006	0.348
RATIO_24H	Mortality rate	0.0109039	0.5335121	−0.3948558	< 0.001	< 0.001	< 0.001	< 0.001
RATIO_24H	Average stay	−0.1650149	0.2168073	−0.4988535	< 0.001	< 0.001	< 0.001	< 0.001
RATIO_24H	Rotation index	0.2074726	−0.1313063	0.5997035	< 0.001	0.034	< 0.001	< 0.001
RATIO_24H	Occupation index	−0.0956397	0.1164279	−0.1538217	0.030	0.088	0.008	< 0.001

## Data Availability

The data supporting the conclusions of this study are available upon reasonable request from the corresponding author. Due to privacy and confidentiality restrictions, not all data can be publicly shared. To access the data, readers may contact the author at (jdfernandez@sescam.jccm.es]. A confidentiality agreement is required to access certain sensitive data.

## Endnotes

^1^Association range: (0–0.19) = very weak; (0.2–0.39) = weak; (0.4–0.59) = moderate; (0.6–0.79) = strong; and (0.8–1) = very strong.

## Supporting Information

Supporting Information for Review and Publication

Supporting Information 1 1. The document ‘2024-039 Juan David Fernández Sánchez .pdf', which is the favorable report issued by the Area's Research Ethics Committee, authorizing the research to be carried out in our hospital.

Supporting Information 2 2. The certificate from the company that performed the translation and linguistic correction of the manuscript into English.
