# Supplementary material for: Relationship Between the Nurse–Patient Ratio and Adverse Events in Conventional Hospitalization Units in a Third-Level Hospital
Source: J Nurs Manag. 2025 Nov 19;2025:8885593. doi: 10.1155/jonm/8885593 (PMC12657084; doi:10.1155/jonm/8885593)
Supplement: Supporting Information 1 — 1. The document ‘2024-039 Juan David Fernández Sánchez .pdf', which is the favorable report issued by the Area's Research Ethics Committee, authorizing the research to be carried out in our hospital. [file 8885593.f1.pdf]

GERENCIA DE ATENCIÓN INTEGRADA DE ALBACETE	**COMITÉ DE ÉTICA DE LA INVESTIGACIÓN CON MEDICAMENTOS**	Informe Conformidad del CEIm

Dª. Karen Nieto Rodríguez, Vicepresidenta del Comité de Ética de la Investigación con Medicamentos de la Gerencia de Atención Integrada de Albacete

INFORMA

Que este Comité en su reunión de fecha 21 de marzo de 2024, Acta 03/2024, ha evaluado el proyecto con código interno Nº 2024-039.

TITULADO: **RELACIÓN ENTRE LA RATIO ENFERMERA-PACIENTE Y LOS EFECTOS ADVERSOS EN UNIDADES DE HOSPITALIZACIÓN CONVENCIONAL EN UN HOSPITAL DE TERCER NIVEL**.

Investigador principal: **D. Juan David Fernández Sánchez.**

Considera que: Se cumplen los requisitos necesarios de idoneidad del proyecto en relación con los objetivos del estudio.

La capacidad del investigador y los medios disponibles son apropiados para llevar a cabo el estudio.

Es adecuado el procedimiento para obtener el consentimiento informado.

Por tanto emite: **INFORME FAVORABLE**.

Este Comité en sus funciones, composición y en los PNT´s cumple con las normas de BPC-CPMP/ICH/135/95 y el Real Decreto 1090/2015 y que su composición actual es la siguiente:

En el caso de que algún miembro participe en un estudio bien como investigador, colaborador o tenga algún conflicto de interés, no habrá participado ni en la evaluación ni en el dictamen de autorización del mismo, en cumplimiento de la normativa legal.

**D. Pedro Abizanda Soler**	PRESIDENTE. Dr. en Medicina. S. Geriatría.
**Dª Karen Nieto Rodríguez**	VICEPRESIDENTA. Dra. en Psiquiatría. S. Psiquiatría
**Dª. Mª Ángeles Lloret Callejo**	SECRETARIA TECNICA. Licenciada en Farmacia. S Farmacia Atención Primaria
**Dª. Raquel Dolores Pérez García**	Vocal representante de los pacientes. Ajeno a la profesión sanitaria
**D. Juan José Núñez Tendero**	Licenciado en Derecho. Ajeno a la profesión sanitaria
**D. Fernando Andrés Pretel**	Ingeniero Técnico Informático de Gestión.
**Dª. Pilar Córcoles Jiménez**	Diplomada en Enfermería. Supervisora de Investigación y Docencia
**Dª. Syonghyun Nam Cha**	Licenciada en Medicina. S. Anatomía Patológica.
**Dª. María Soledad Fernández de Córdoba**	Dra. en Medicina. S. Cirugía Pediátrica
**D. Ignacio Párraga Martínez**	Dr. en Medicina. Centro de Salud Zona VIII
**D. Manuel Gerónimo Pardo**	Dr. en Medicina. S. Farmacología Clínica
**D. Carlos Marcilla Vázquez**	Licenciado en Medicina. S. Pediatría
**D. Raúl Godoy Mayoral**	Dr. en Medicina. S. Neumología
**D. Francisco Javier Callejas González**	Dr. en Medicina. S. Neumología
**Dª. María Rosa Ortiz Navarro**	Licenciada en Farmacia. Farmacia Hospitalaria
**Dª. María Munera Valero**	Licenciada en Medicina. S. Medicina Intensiva
**D. Rubén Alcantud Córcoles**	Licenciado en Medicina. S. Geriatría
**Dª María Dolores Pérez Carrión**	Vocal representante de la UCLM
**Dª Eva María Galán Moya**	Vocal representante de la UCLM
**D. Eduardo Tebar Martínez**	Vocal, representante FIR

Albacete, 21 de marzo de 2024

NIETO RODRIGUEZ KAREN NATACHA - 49426026S

Firmado digitalmente por NIETO RODRIGUEZ KAREN NATACHA - 49426026S
Fecha: 2024.04.03 10:19:02 +02'00'

Fdo.: Karen Nieto Rodríguez
