# Supplementary figures and images for: Relationship Between the Nurse–Patient Ratio and Adverse Events in Conventional Hospitalization Units in a Third-Level Hospital
Source: J Nurs Manag. 2025 Nov 19;2025:8885593. doi: 10.1155/jonm/8885593 (PMC12657084; doi:10.1155/jonm/8885593)

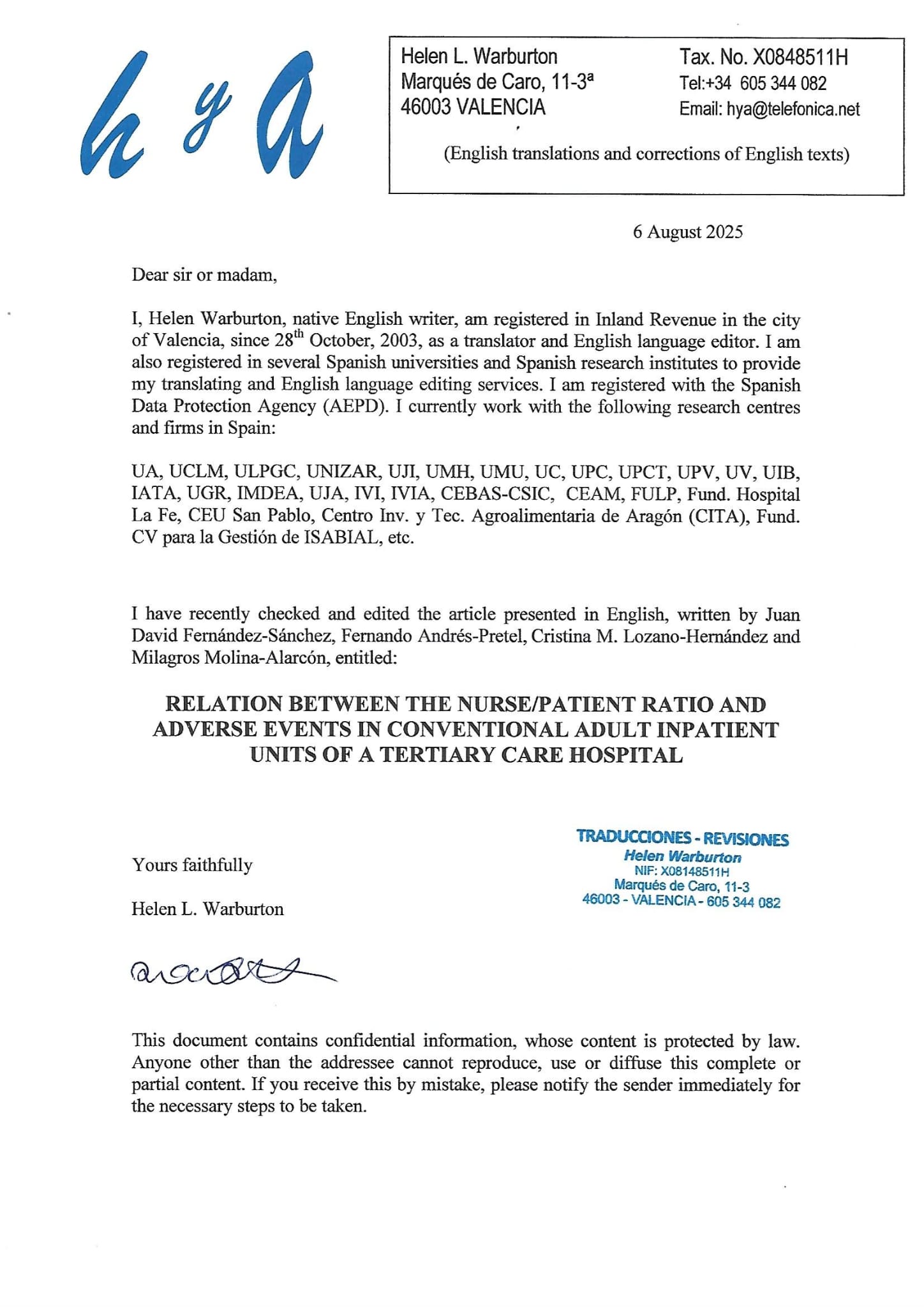

Supplement: Supporting Information 2 — 2. The certificate from the company that performed the translation and linguistic correction of the manuscript into English. [file 8885593.f2.jpg]
